# Supplementary material for: Extreme–ultraviolet high–harmonic generation in liquids
Source: Nat Commun. 2018 Sep 13;9:3723. doi: 10.1038/s41467-018-06040-4 (PMC6137105; doi:10.1038/s41467-018-06040-4)
Supplement: Supplementary file 1 — Supplementary Information [file 41467_2018_6040_MOESM1_ESM.pdf]

Supplementary information for
Extreme–ultraviolet high–harmonic generation in liquids

Tran Trung Luu*+, Zhong Yin+, Arohi Jain, Thomas Gaumnitz, Yoann Pertot, Jun Ma, and Hans Jakob Wörner†
Laboratorium für Physikalische Chemie, ETH Zürich, 8093 Zürich, Switzerland

(Dated: July 31, 2018)

SUPPLEMENTARY NOTE 1: TWO-SOURCE INTEREFEROMETRY REVEALS COHERENCE PROPERTIES OF HHG FROM LIQUIDS

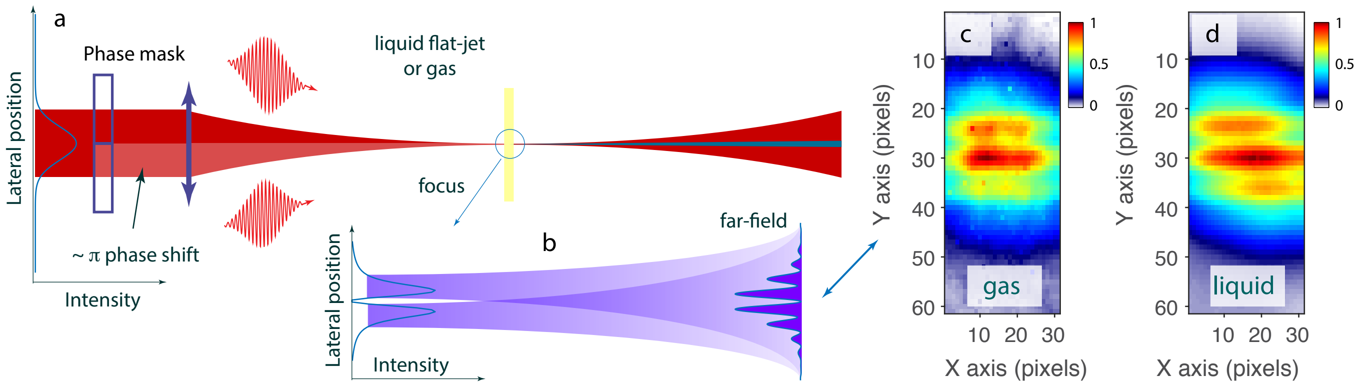

Supplementary Figure 1. **Coherence properties of HHG from liquids compared to gas**. **a**, Experimental scheme for two–source interferometry consisting of a phase–mask, a focusing mirror, a liquid flat microjet and a detection system. The cross–cut of the beam profiles in the far field and at the focus are shown in **b**. Harmonics are generated at the peak intensity and they interfere in the far field. **c, d**, Recorded raw images of the spatio–spectrally resolved interference patterns for gas and liquid samples, respectively. The images are taken at the same photon energy corresponding to H11.

SUPPLEMENTARY NOTE 2: EUV HHG FLUX MEASUREMENT

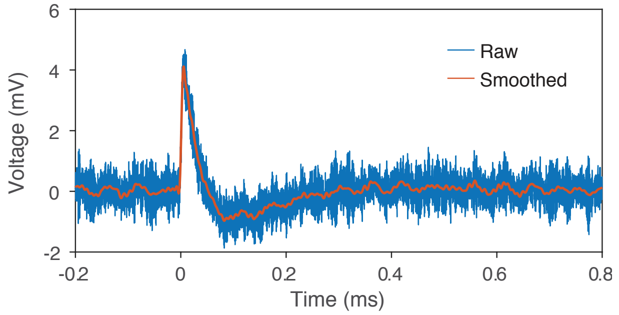

Supplementary Figure 2. **Quantitative measurement of photon flux of HHG from liquids**. Signal recorded from an aluminium–coated XUV photodiode after passage through a charge amplifier. The liquid sample is ethanol.

We quantified the photon flux generated in our experiments by replacing the MCP/phosphor-screen assembly with an aluminium-coated XUV photodiode (AXUV100Al - OptoDiode) and recorded the electronic signal from the photodiode upon exposure to EUV HHG from liquids. Supplementary figure 2 shows a typical temporal signal observed in a flux measurement. Calibration of these measured data using calibrated spectra, photodiode response, grating efficiency, and charge-amplifier efficiency results in the numbers reported in the main text. The largest sources of error are the grating efficiency (30%) and charge-amplifier efficiency (20%). Including other types of errors results in an overall error of 70%.

SUPPLEMENTARY NOTE 3: LOCAL STRUCTURE IN LIQUID WATER

There are two important reasons that suggest the use of the semiconductor Bloch equations (SBE), originally developed for periodic systems, for calculating the microscopic response of water and alcohols. First, our experimental data on the scaling of the cut-off photon energy with peak electric field is a linear function. This observation agrees with previous results on solids [1–3]. Second, although they lack long-range order, liquid water and alcohols display a

strong local order imposed by the hydrogen bonding network [4–8]. In particular, the O-O pair-correlation function of

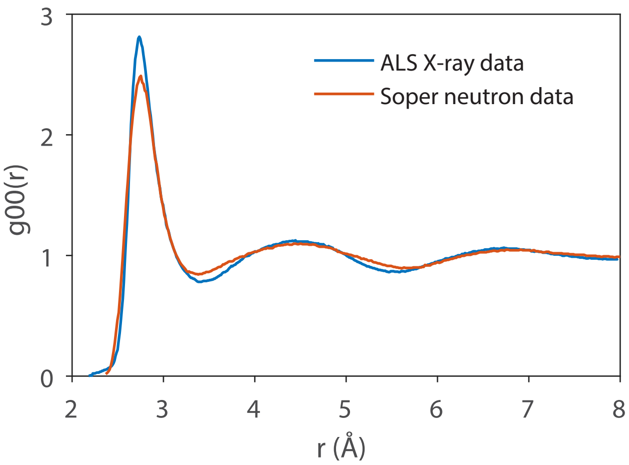

Supplementary Figure 3. **Comparison of pair correlation function of oxygen-oxygen distance from X-ray and neutron scattering experiments extracted from [6, 7].**

liquid water obtained from neutron and X-ray scattering measurements (Supplementary Fig. 3) supports the view of a strong local ordering of water molecules, with a narrowly defined first oxygen-oxygen distance of ≈ 2.8 Å. This data further shows the presence of two additional maxima (at ≈ 4.4 Å and ≈ 6.8 Å), which correspond to two additional hydration shells. Thus microscopically, the existence of partial local order supports the use of the SBE approach to simulate the microscopic response of liquid water.

SUPPLEMENTARY REFERENCES

[1] Shambhu Ghimire, Anthony D. DiChiara, Emily Sistrunk, Pierre Agostini, Louis F. DiMauro, and David A. Reis, "Observation of high-order harmonic generation in a bulk crystal," Nature Physics **7**, 138–141 (2011).

[2] T T Luu, M Garg, S Yu Kruchinin, A Moulet, M Th Hassan, and E Goulielmakis, "Extreme ultraviolet high-harmonic spectroscopy of solids," Nature **521**, 498–502 (2015).

[3] Georges Ndabashimiye, Shambhu Ghimire, Mengxi Wu, Dana A. Browne, Kenneth J. Schafer, Mette B. Gaarde, and David A. Reis, "Solid-state harmonics beyond the atomic limit," Nature **534**, 520–523 (2016).

[4] Ph. Wernet, D. Nordlund, U. Bergmann, M. Cavalleri, M. Odelius, H. Ogasawara, L.Å.Naslund, T. K. Hirsch, L. Ojamae, P. Glatzel, L. G. M. Pettersson, and A. Nilsson, "The Structure of the First Coordination Shell in Liquid Water," Science **304**, 995–999 (2004).

[5] G. N. I. Clark, G. L. Hura, J. Teixeira, A. K. Soper, and T. Head-Gordon, "Small-angle scattering and the structure of ambient liquid water," Proceedings of the National Academy of Sciences **107**, 14003–14007 (2010).

[6] Teresa Head-Gordon and Greg Hura, "Water Structure from Scattering Experiments and Simulation," Chemical Reviews **102**, 2651–2670 (2002).

[7] A. K. Soper, "Joint structure refinement of x-ray and neutron diffraction data on disordered materials: Application to liquid water," Journal of Physics Condensed Matter **19** (2007), 10.1088/0953-8984/19/33/335206.

[8] John Russo and Hajime Tanaka, "Understanding water's anomalies with locally favoured structures," Nature Communications **5**, 1–11 (2014), arXiv:1308.4231.
