# Supplementary material for: Extreme–ultraviolet high–harmonic generation in liquids
Source: Nat Commun. 2018 Sep 13;9:3723. doi: 10.1038/s41467-018-06040-4 (PMC6137105; doi:10.1038/s41467-018-06040-4)
Supplement: Supplementary file 3 — Description of Additional Supplementary Files [file 41467_2018_6040_MOESM3_ESM.pdf]

Description of Additional Supplementary Files

File Name: Supplementary Movie 1
Description: **Monitoring ultrashort laser pulses – flat-microjet liquid interaction**: A movie taken from an off-axis perspective of the flat microjet. The whole flat microjet is moved in the vertical direction while all other experimental conditions including the laser beam are kept unchanged. Once the flat microjet is high enough, one can observe clearly the green spot in the middle of the flat microjet which is the interaction area. The surrounding green light is the scattering of the third harmonic on the plume of liquid micro-droplets.

File Name: Supplementary Movie 2
Description: **Complete separation of HHG from liquid and gas:** A recorded series of raw images taken from the 2D detector with an energy-calibrated axis. The Y pixels denote the vertical axis of the detector. The images are taken for different vertical positions of the flat jet, corresponding to the Supplementary Movie 1. The offset is arbitrary. One can see the maximum separation between HHG from liquid water (bright spots at the bottom) and HHG from gas-phase water (faint spots at the top, and at the end of the movie) happens in the beginning of the scan, i.e. at the top of the flat jet where the curvature or wedge angle is maximized. The integration time is set to 30 ms to avoid saturation of the HHG signal from the liquid phase. Therefore the signal to noise ratio is limited for the gas-phase contribution.
